# Labelling effects and adolescent responses to peers with depression: an experimental investigation

**DOI:** 10.1186/s12888-017-1389-9

**Published:** 2017-06-24

**Authors:** Louise Dolphin, Eilis Hennessy

**Affiliations:** School of Psychology, University College Dublin, Belfield, Dublin 4, Dublin, Ireland

**Keywords:** Labelling, Stigma, Gender, Peers

## Abstract

**Background:**

The impact of illness labels on the stigma experiences of individuals with mental health problems is a matter of ongoing debate. Some argue that labels have a negative influence on judgments and should be avoided in favour of information emphasising the existence of a continuum of mental health/illness. Others believe that behavioral symptoms are more powerful influencers of stigma than labels. The phenomenon has received little attention in adolescent research, despite the critical importance of the peer group at this developmental stage. This study employs a novel experimental design to examine the impact of the depression label and continuum information on adolescents’ responses to peers with depression.

**Methods:**

Participants were 156 adolescents, 76 male, 80 female (*M* = 16.25 years; *SD* = .361), assigned to one of three conditions (Control, Label, Continuum). Participants respond to four audio-visual vignette characters (two clinically depressed) on three occasions. Outcome measures included judgment of the mental health of the vignette characters and emotional responses to them.

**Results:**

Neither the provision of a depression label or continuum information influenced perceptions of the mental health of the characters in the audio-visual vignettes or participants’ emotional responses to them.

**Conclusion:**

The findings have implications for the design of interventions to combat depression stigma with adolescents. Interventions should not necessarily target perceptions of psychiatric labels, but rather perceptions of symptomatic behaviour.

## Background

Some adolescents report that the depression label has a negative effect on their sense of self and their view of the future, contributing to an illness identity that hinders recovery [1]. Fear of labels and anticipation of stigma is a barrier to adolescents’ help-seeking [2]. However, few studies have tried to understand such stigma by investigating the adolescent peer group’s response to depression labels.

### Labels and perception

Early perception research [3] established that the application of category labels distorts the perception of simple objects by increasing the apparent differences between stimuli belonging to different classes (“between-category accentuation”; BCA), and by increasing the apparent similarity of stimuli belonging to the same class (“within-category assimilation”; WCA), a phenomenon replicated in numerous object perception studies [4, 5]. Social psychologists have also investigated how category labels structure perceptions of social groups. Category labels provide a perceiver with a resource to navigate the social environment, serving as an information-processing lens for interpreting and integrating social information [6]. However, as with object perception, labels can induce categorical representations that reduce perceived differences between members of the same group while exaggerating perceived differences between members of different groups [7, 8].

Research on category labels is of interest to those studying the effects of mental disorder labels on subsequent stigma responses, because diagnostic classifications augment public perceptions of the discreteness and differentness of people with mental disorders [9]. Diagnosis adds to the perception that those who have received a mental disorder label are a meaningful or unified entity [10].

### Depression labelling and emotional responses

While the association between labels and stereotyping is well documented [11], the impact of labels on emotional reactions associated with stigma is poorly understood. Some research [12] has found that adults who label an individual with depression as “mentally ill” were less likely to react with anger, but this label did not significantly predict sympathy or fear. Other researchers have found that agreement with a mental illness label for depression does not reduce anger but, paradoxically, is associated with an increase in both sympathy and fear [13]. Emotions of anger, sympathy, irritation, fear, and anxiety can be detected by a person who is stigmatised, but these emotional responses can also shape the subsequent behaviour of the stigmatiser [14]. There is a widely cited link between emotions of sympathy, anger and fear, and behavioural intentions toward persons with mental disorders including depression [12, 15, 16]. However, emotional reactions are frequently overlooked in the stigma literature - particularly in the adolescent stigma literature. No previous experimental studies have manipulated the depression label to assess its impact on emotional reactions.

### Continuum beliefs and mental health

An alternative way to understand diagnosis is dimensionally rather than categorically [17]. Rather than assign someone to a class of people with similar symptoms, course, and disabilities, dimensional diagnosis seeks to describe a person’s profile of symptoms on a continuum that includes normal life [9].

Schomerus and colleagues [13] argue that many people do not fulfil the criteria for a mental disorder but still experience various psychiatric symptoms to different degrees. They propose that anti-stigma messages could foster the perception that a person with a mental disorder is someone like us, and that his/her experiences resemble our own. They established that continuum beliefs about depression are associated with reduced fear and increased positive emotions. Their findings also stress that experimental studies manipulating diagnostic labels and continuum explanations of mental disorder are necessary to further understand these relationships.

In light of the fact that a taxometric analysis of depression in children and adolescents concluded that the latent structure of depression was dimensional, not categorical [18], this study is interested in how this information would affect adolescents’ reactions to peers with depression. Of note, individuals make more use of category boundaries than meaningful continuous information, indicating the disproportionate power of labels in influencing perception and judgment [19].

### The present study

Recognising and labelling depression in survey based studies produces mixed stigma responses from adolescents [20, 21, 22]. This study explores the effects of experimentally manipulating depression labels and continuum information on adolescent emotional (stigma) responses and mental health evaluations (global judgment) of hypothetical male peers with depression, as male adolescents with depression evoke stronger stigma responses from peers than their female counterparts [23, 24].

Improved understanding of the effect of clinical labels on reactions to adolescents who are depressed, has important implications for the design of mental health literacy and anti-stigma interventions. Such interventions are increasingly recognised as important contributors to improving health outcomes (for example, by promoting help seeking) [25] and research by Chisholm [26] demonstrates that it is possible to use school-based interventions to change attitudes toward people with serious mental health problems.

### Hypotheses

1. A depression label will produce BCA effects on participants’ judgments and emotional reactions to a series of male peers presented with varying symptoms of depression. These effects will be most pronounced in the group provided with a label, and lower in the group provided with continuum information. No effects are anticipated in the control group.
2. A depression label will produce WCA effects on participants’ judgments and emotional reactions to a series of male peers presented with varying symptoms of depression. These effects will be most pronounced in the group provided with a label, and lower in the group provided with continuum information. No effects are anticipated in the control group.
3. BCA and WCA effects will be significantly reduced once the depression label is removed. This is in line with previous research which found that when labels are removed and no longer present, the effect of labels, although diminished, persisted [19].
4. Participant gender will influence responses. Previous research has found that male and female adolescents respond differently to male peers with depression [23] suggesting the need to included gender as a variable in this research.

## Methods

### Research design and determining the sample size

An experimental design with three experimental conditions (Control, Label, and Continuum) was employed. Participants in each condition were tested on three occasions (Time 1, Time 2, Time 3). Formal a priori power calculations were conducted using G*Power 3.1 [27] to determine sample size. G*Power estimated that 144 participants would be required to carry out the proposed analysis (power = 0.8, α =0.05, effect size = 0.25).

### Participants

Participants were 156 adolescents (76 male, 80 female), age range 14.83 to 17.16 years (M = 16.25; SD = .361). Participants were recruited through their schools in the Dublin area. Four schools participated (two mixed schools, two single sex schools). Participants were assigned to one of the three conditions – Control (*n* = 52; 24 male, 28 female), Label (*n* = 52; 28 male, 24 female) or Continuum (*n* = 52; 24 male, 28 female). Written parental consent and participant verbal assent was obtained from all participants. There was a response rate of 46%. Ethical approval for this study was granted from the Human Research Ethics Committee-Humanities of the corresponding author’s university.

### Materials

**Fig. 1:**
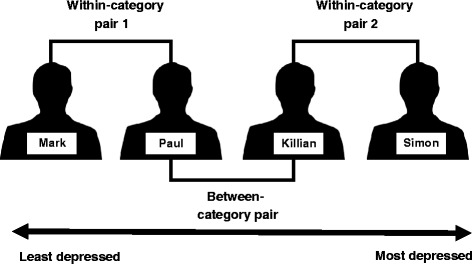
Categorisation index: Between-category and within-category pairs

Stimulus material were four audiovisual vignettes depicting two characters (Mark and Paul) who did not meet the DSM-5 [28] diagnostic criteria for Major Depressive Disorder, and two characters (Killian and Simon) who met the DSM-5 diagnostic criteria for Major Depressive Disorder (see Fig. 1). In the scripts written for the vignettes, the characters give verbal descriptions of their symptoms: Mark shows very little evidence of depressive symptoms, Paul shows evidence of depressive symptomatology though not severe enough to meet the DSM-5 diagnostic criteria, Killian and Simon both meet the DSM-5 diagnostic criteria but the Killian character is less impaired than the Simon character. These scripts were revised by a convenience sample of five psychologists (two qualified clinical psychologists and three trainee clinical psychologists who had completed their child and adolescent clinical placement). See Appendix for revised vignette scripts. Male undergraduate students volunteered as actors for the recording of audiovisual vignettes. The average length of a vignette was 1 min and 17 s.

### Questionnaire

Participants responded to the audiovisual vignettes in a questionnaire pack.

Emotional reactions towards the characters in the audiovisual vignettes were measured based on Angermeyer and Matschinger’s [29] identification of three types of emotional reactions towards individuals with mental illness: aggressive emotions (e.g. anger/ irritation), feelings of anxiety (e.g. discomfort/ uneasiness) and prosocial reactions (e.g. sympathy/ pity). Participants were asked to rate three emotional reactions: one for each of these dimensions on a 6-point Likert scale ranging from 1 (strongly disagree) to 6 (strongly agree).

Mental Health Evaluation score: Participants were asked to rate the mental health of each vignette character (e.g. “How would you rate Simon’s mental health?”) on a 6-point Likert scale ranging from 1 (very poor) to 6 (very good). The purpose of the mental health evaluation (MHE) question was to include a global, objective question about the character’s functioning (as opposed to the subjective nature of emotional responses).

Evaluation of actors and endorsement of continuum: Following a short debrief, participants were asked to complete a final page of questions about how realistic and likeable each actor was, alongside a question about how much they endorsed the statement “We are all sometimes like Simon or Killian, it’s just a question of how extreme that state is”. These responses were measured using 6 point Likert scales.

### Procedure

Participants were assigned to one of the three conditions – Control, Label or Continuum. It should be noted that while school and condition were not confounded, intact groups (class groups) were assigned to conditions within the schools. Testing took approximately 40 min. Participants were not explicitly told that the study was about depression. They were informed that they would watch four short videos and would respond to each character in their questionnaire pack. To control for order of vignette presentation acting as a confounding variable, a Latin Square system [30] was used to determine the order of presentation, thereby counterbalancing the presentation of vignettes. Participants responded to each vignette directly after viewing, to reduce load on memory and reduce bias judgments toward vignettes presented at the beginning or end of the sequence.

Time 1: Participants watched each audiovisual vignette and responded to each character in their questionnaire pack directly after viewing the vignette. They then completed a 2 min word search as a breaker task between Time 1 and Time 2. Following this, Time 1 responses were collected.

Time 2: Participants watched the vignettes again. Participants in the Control Group were not given any additional information. Participants in the Label Group were told “This time, it is important to note that Simon and Killian would receive a diagnosis of clinical depression from a psychiatrist or clinical psychologist, and that Mark and Paul would not receive a diagnosis of clinical depression from a psychiatrist or a clinical psychologist.” Participants in the Continuum Group were told “This time, it is important to note that Simon and Killian would receive a diagnosis of clinical depression from a psychiatrist or clinical psychologist and Mark and Paul would not. However, recent evidence from an important psychology journal tells us that anyone can experience symptoms like these, it is just a matter of how extreme they are. For example, while Paul would not receive a diagnosis of clinical depression, he is still experiencing certain, less extreme symptoms of depression.” The researcher then played the vignettes again, pausing after each, to allow participants respond. Upon completion, participants were asked to complete another 2 min timed word search (breaker task) between Time 2 and Time 3. Responses at Time 2 were collected.

Time 3: Participants watched the vignettes for a third time with the same instructions as at Time 1 (i.e. no references to labels or continuum) and responded to each character. Time 3 responses were then collected from participants.

Once responses at Time 3 were collected, participants were thanked for their participation and a short debrief was carried out, during which participants were informed that the characters in the vignettes were actors who had volunteered to take part. Participants in the continuum condition were informed “While a new area of research indicates that many people in the population experience some symptoms of depression, it is still important to take these symptoms seriously and it does not make a diagnosis of clinical depression less meaningful.” All participants then responded to questions pertaining to how likeable and realistic each actor was, alongside how much participant’s endorsed a continuum belief about depression.

### Analysis plan

The plan for analysis was to *a)* calculate a BCA and WCA score for each dependent variable (anger, sympathy, discomfort, MHE) at each time point; *b)* compare the three groups on BCA and WCA baseline scores; *c)* conduct a series of (eight) 2x3x3 mixed between-within subjects ANOVAs to examine the impact of gender (between subjects, two levels: male, female), condition (between subjects, three levels: Control Group, Label Group, Continuum Group) and time (within-subjects, three levels: Time 1, Time 2, and Time 3) on the four BCA scores, and the four WCA scores.

A second set of analyses was also planned, using ANOVA of change scores. To compute change scores, we subtracted the Time 1 score from the Time 2 score (change score 1) and the Time 2 score from the Time 3 score (change 2 score) separately for each group, at each time point. Two-way ANOVAs were conducted on the change scores from Time 1 to Time 2, and Time 2 to Time 3. This supplementary analyses provided the same pattern of results as the mixed between-within subjects ANOVAs and thus, the former analyses are presented in this paper.

## Results

### Data management and treatment of missing data

Data were entered into Statistical Package for the Social Sciences (SPSS) Version 20. One participant had over 10% missing data and was thus removed from further analyses. Missing data for remaining participants (*n* = 3 with 1.7% missing data in all cases) were imputed using were imputed using the Expectation Maximization algorithm [31].

### Preliminary analyses

Preliminary analyses were conducted to assess differences between the three groups in terms of age and evaluation of vignette characters. A one-way ANOVA revealed no difference in age across the three groups, *F*(2, 155) = 1.43, *p* = .241. In addition there was no difference across groups in terms of how realistic or likeable participants perceived the vignette characters to be (realistic/Mark *F*(2, 153) = 1.72, *p* = .182, realistic/Paul *F*(2, 153) = 1.18, *p* = .308, realistic/Killian *F*(2, 153) = .498, *p* = .609, realistic/Simon *F*(2, 153) = 2.66, *p* = .073, likeable/Mark *F*(2, 153) = 1.16, *p* = .315, likeable/Paul *F*(2, 153) = .750, *p* = .474, likeable/Killian *F*(2, 153) = .450, *p* = .639, likeable/Simon *F*(2, 153) = .450, *p* = .639)

As a manipulation check at the debrief stage, participants were also asked for their agreement with a continuum statement: “How much do you agree with the statement “We are all sometimes like Simon or Killian, it’s just a question of how extreme that state is””. They responded on a scale from 1 (strongly disagree) to 6 (strongly agree). Descriptive statistics indicated that participants in the Continuum Group accepted/endorsed the validity of the continuum information (*M* = 4.61; *SD* = 1.38).

### Data reduction

Raw data consisted of participants’ responses (MHE and emotional reactions: anger, sympathy, discomfort) to each of the four vignette characters at Time 1, Time 2, and Time 3. From the raw data, “between category accentuation” (BCA) and “within category assimilation” (WCA) scores were calculated, guided by Foroni and Rothbart’s [19] data reduction analysis. The BCA scores comprised the absolute difference between responses to Killian and Paul. Only Killian and Paul were chosen for the BCA as their vignettes are directly on either side of the depression label boundary. We believe to add all four characters into this (i.e. add the difference between Mark and Killian, and Paul and Simon), would dilute the BCA result.

This produced a score ranging from 0 (no difference) to 5 (large difference). As there are two within-category pairs (i.e. Mark & Paul, and Killian & Simon), absolute difference scores for these pairs were averaged to create the WCA scores. This also produced a score ranging from 0 (no difference) to 5 (large difference). See Fig. 1 for a visual representation of between-category and within-category pairs. See Table 1 for descriptive statistics for BCA and WCA scores.

**Table 1:** Descriptive statistics for between category accentuation and within category assimilation scores

Variable	Condition	Time	Between category accentuation			Within category assimilation		
			Min-Max	*M*	*SD*	Min-Max	*M*	*SD*
Mental health	Control	1	0–4	1.55	1.01	0.00–3.00	1.28	0.58
		2	0–4	1.75	1.08	0.00–3.00	1.25	0.62
		3	0–4	1.90	1.03	0.00–3.00	1.14	0.67
Mental health	Label	1	0–4	1.71	0.98	0.50–3.00	1.34	0.58
		2	0–4	1.90	1.05	0.50–2.50	1.35	0.60
		3	0–4	1.94	1.05	0.50–3.50	1.41	0.66
Mental health	Continuum	1	0–4	1.59	0.91	0.00–2.50	1.24	0.54
		2	0–5	1.90	1.10	0.00–3.50	1.16	0.67
		3	0–5	1.82	1.11	0.00–3.00	1.25	0.69
Sympathy	Control	1	0–4	1.50	1.03	0.00–3.00	1.20	0.67
		2	0–5	1.59	1.25	0.00–3.50	1.17	0.70
		3	0–5	1.40	1.27	0.00–4.00	1.19	0.77
Sympathy	Label	1	0–4	1.76	1.04	0.00–4.00	1.27	0.88
		2	0–5	1.50	1.22	0.00–3.50	1.15	0.73
		3	0–5	1.50	1.21	0.00–3.00	1.22	0.74
Sympathy	Continuum	1	0–5	1.55	1.12	0.00–2.50	1.23	0.69
		2	0–5	1.50	1.40	0.00–4.50	1.21	0.93
		3	0–5	1.48	1.32	0.00–3.00	1.23	0.85
Anger	Control	1	0–2	0.63	0.79	0.00–2.50	0.61	0.75
		2	0–4	0.62	0.95	0.00–2.50	0.47	0.64
		3	0–3	0.56	0.83	0.00–3.00	0.65	0.75
Anger	Label	1	0–3	0.81	1.01	0.00–2.50	0.60	0.61
		2	0–5	0.63	0.95	0.00–2.50	0.58	0.62
		3	0–4	0.60	0.89	0.00–4.00	0.52	0.75
Anger	Continuum	1	0–3	0.79	0.89	0.00–4.00	0.93	0.83
		2	0–5	1.11	1.27	0.00–5.00	0.89	1.06
		3	0–5	0.88	1.14	0.00–4.00	0.97	1.01
Discomfort	Control	1	0–4	1.38	1.14	0.00–3.50	0.76	0.80
		2	0–4	1.17	1.09	0.00–2.50	0.60	0.56
		3	0–4	0.92	1.20	0.00–2.50	0.78	0.63
Discomfort	Label	1	0–4	1.32	1.04	0.00–3.50	1.15	0.97
		2	0–4	1.13	1.15	0.00–3.50	1.05	0.82
		3	0–4	1.17	1.07	0.00–2.50	0.83	0.73
Discomfort	Continuum	1	0–5	1.38	1.41	0.00–2.50	1.09	0.85
		2	0–5	1.59	1.53	0.00–5.00	1.10	0.99
		3	0–5	1.46	1.48	0.00–3.50	0.88	0.82

*M* mean, *SD* standard deviation

### Assessment of baseline differences between groups

Differences in baseline (Time 1) variables were assessed to ensure that groups were comparable. A series of eight one-way ANOVAs were employed to assess baseline scores for the four BCA scores and the four WCA scores. The false discovery rate was used to control for Type 1 error associated with making multiple comparisons [32]. Using these adjusted *p* values (between < .006 and < .05 for the 8 comparisons), the groups did not differ on any baseline score.

### ANOVA analyses

Eight 3 × 3 × 2 mixed between-within subjects ANOVAs were carried out to examine the impact of condition (between subjects, three levels: Control Group, Label Group, Continuum Group), time (within-subjects, three levels: Time 1, Time 2, and Time 3), and participant gender (between subjects, two levels: male female), on the four BCA scores, and the four WCA scores.

The initial focus of our analysis for each hypothesis was on the three-way interaction to determine, at the outset, whether a condition*time interaction varied as a function of gender on the basis of findings of previous research [23, 24]. In relation to our first three hypotheses, our focus was on the two-way interaction between condition*time. The primary evidence for categorisation is present in the difference between BCA and WCA scores as we move from an uncategorised state (Time 1) to a categorised state (Time 2). It was hypothesised that a significant Condition*Time interaction would capture that this effect is most prominent in the Label Group, followed by the Continuum Group, and non-existent in the Control Group. It was hypothesised that a significant Condition*Time interaction would also indicate that this effect is reduced at Time 3 when labels are no longer present. The false discovery rate was again used to adjust significance levels [32] yielding adjusted *p* values of between < .006 and < .05). Only statistics that are significant at the adjusted levels are reported here.

One three-way interaction was significant (WCA sympathy), *F*(4, 300) = 3.52, *p* = .008. The dataset was thus split for male and female participants and the Condition*Time interaction was interpreted. However, there was not a significant Time*Condition interaction for either males or females, nor was there a significant main effect for Time, or Condition. No significant interactions were observed between Condition*Time, Condition*Gender, or Time*Gender on any of the eight dependent variables. Finally, on inspection of the main effects, three significant main effects were identified. For BCA mental health scores, there was a main effect for Time, *F*(2, 149) = 4.07, *p* = .019. Tukey post hoc comparison indicates that, regardless of gender and condition, all participants had marginally lower (*p* = .06) BCA Mental Health scores at Time 1 (*M* = 1.62) compared to Time 2 (*M* = 1.84) and significantly lower (*p* = .016) than Time 3 (*M* = 1.89). For WCA anger scores there was a significant main effect for condition, *F*(2, 150) = 5.44, *p* = .005. Tukey post hoc analysis indicated that regardless of Time and Gender, the Continuum Group had significantly higher scores (*M* = .936) than the Control Group (*M* = .577) and the Label Group (*M* = .567), *p* = .016 and .012 respectively. For WCA discomfort scores, there was a main effect for Condition, *F*(2, 150) = 4.29, *p* = .015. Tukey post hoc analysis indicated that the Control Group had significantly lower WCA discomfort scores (*M* = .710) than the Label Group (*M* = 1.01) or the Continuum Group (*M* = 1.02), *p* = .034 and .026 respectively.

#### Hypothesis 1:

That a depression label will produce BCA effects on participants’ judgments and emotional reactions to a series of male peers presented with varying symptoms of depression, and that this effect will be reduced where continuum information is provided, was not supported. Evidence for this conclusion is based on the non-significant Condition*Time interactions.

#### Hypotheses 2:

That a depression label will produce WCA effects on participants’ judgments and emotional reactions to a series of male peers presented with varying symptoms of depression, and that this effect will be reduced where continuum information is provided, was not supported. Although there was a significant three-way interaction for WCA sympathy scores, the Condition*Time interaction did not vary as a function of participant gender. For WCA anger scores there was a significant main effect for condition indicating that, regardless of Time and Gender, the Continuum Group had higher scores than the Control Group and the Label Group. For WCA discomfort scores, there was a main effect of Condition indicating that the Control Group had lower WCA discomfort scores than the Label Group or the Continuum Group. However, as there was no interaction between Condition*Time, it cannot be inferred that these relationships emerged as a function of depression labels.

#### Hypothesis 3:

That labelling effects will be significantly reduced once the depression label is removed (at Time 3). As labelling effects were not established at Time 2, Hypothesis 3 was deemed void.

#### Hypothesis 4:

That participant gender will influence labelling effects, was not supported.

## Discussion

The aim of this study was to experimentally investigate the impact of depression labels and continuum information on adolescents’ responses to peers with varying symptoms of depression. Previous social cognitive research indicates that the imposition of category labels encourages individuals to ignore the large variation within a category, and exaggerate the differences between individuals who are barely on opposite sides of a category boundary [19]. To our knowledge this is the first study to experimentally manipulate both the depression label and continuum information to measure reactions to mental health diagnoses, as advocated by Schomerus and colleagues [13]. The results provide valuable data on the effects of categorical versus continuum information which may inform anti-stigma interventions with this age group. Results will also add to the debate among labelling theorists about whether a depression label or an adolescent’s (unlabelled) depressed symptomology has a stronger influence on the judgments and reactions of their peers.

Results indicate that neither the provision of depression labels nor continuum information produced categorisation effects in participants’ responses. These results provide important information regarding the impact of depression labels on judgments of, and responses to, adolescents with depression. Using a novel methodological and statistical approach, results indicate that symptomology, rather than depression labels influence adolescents’ reactions. This is in line with the work of Hinshaw [33], who postulates that reactions to the behaviors associated with mental health difficulties may be equally or even more important than the label.

While results are at odds with the findings of category perception research involving both objects [3–5] and social stimuli [19, 34], they are not directly comparable because we *a)* utilised audio-visual as opposed to visual stimuli *b)* did not measure similarity as the dependent variable [3, 19]; but rather the consequence of the cognitive judgment on perceptions of, and reactions to depression. Although the use of the audio-visual vignettes hampers comparability to studies that have used visual stimuli we believe that their use has strengthened the ecological validity of the research. Experimental research in the medical literature indicates that video vignettes allow effective manipulation, are perceived as realistic, and enable observers to immerse themselves in the situation depicted [35]. Thus, they can yield valid and informative results. Descriptive analysis indicated that participants rated all vignette characters as realistic in this study.

### Limitations

The application of our findings to real world situations must be tentative. As Link and colleagues [36] outline - the extent to which an experiment reflects social processes outside of a constructed situation is questionable. They argue that vignettes present relatively concrete, specific information. In daily interaction, information is likely to come from different, perhaps contradictory sources, thereby providing a more ambiguous picture of events than vignettes offer. Labelling effects may be different under such conditions of uncertainty. Because of the exploratory nature of our study we also chose to use only males in our vignettes, based on previous research findings that adolescents’ responses to males with symptoms of depression are more negative than towards females [23]. However, it would be important to determine whether labels also lack significance for female characters.

Another methodological limitation is that participants were not individually randomised to each condition. As with the majority of social research, this study was structured like a pretest-posttest-follow-up randomised experiment, but it lacks the key feature of randomised designs - individual random assignment, as intact class groups were assigned to conditions within schools. In addition, due to the requirement to conduct the experiment in one class period (40 min), the breaker tasks between time points (a timed 2 min word search) were short, potentially compromising participants’ ability to fully disengage from the previous time point. A further consequence of our limited time was the decision to use single-item questions for all dependent variables. However, single-items have ethical and practical advantages over multi-item measures as they reduce participant fatigue and are less monotonous [37], a key issue when using items repeatedly in an experimental study.

This study only looks at the impact of depression label versus continuum information on one aspect of stigma – emotional reactions. Stigma is a multidimensional construct [10] and these findings are not generalisable to other aspects of stigma (e.g. stereotyping). Finally, as data on participants’ behavioural and emotional profiles and their familiarity with depression in friends and family were not collected, the sample in this study was potentially a mixed pool of participants, whereby some may have had personal experience with depression, potentially confounding results.

### Implications

This study provides information to guide labelling theorists specifically interested in adolescent depression stigma. Results indicate that adolescents respond consistently to symptoms of depressed behaviour in male peers and these responses are not altered by the provision of a depression label, supports theorists such as Gove [38]. On a practical level, findings are of interest to those designing interventions to combat depression stigma with adolescents. Interventions should not necessarily target perceptions of psychiatric labels, but rather perceptions of symptomatic behaviour.

### Future research

Future researchers could create a computerised version of this experiment [19] which would allow for true randomisation of participant to condition. This method of exploring labelling effects, influenced by category perception research, could be employed to investigate reactions to different mental health disorders. As Hogg and Williams [39] outline, categorisation effects are amplified when the categorisation is personally relevant to the perceiver. Tajfel [40] believed that this effect was even stronger for the perception of people as opposed to objects because self is involved; the perceiver usually falls within one category. It is thus imperative that future research considers the implications of self-identification on adolescents’ responses to peers with depression. Finally, given the limited generalisability of these findings to only male adolescents, and to only one component of stigma (emotional reactions), future researchers should continue to investigate the effect of labels and continuum information on adolescent depression stigma.

## Conclusions

This study found no evidence that adolescents’ responses to male peers who have symptoms of depression are influenced by a label of clinical depression. This finding supports theorists who argue that stigma is associated with symptoms, rather than psychiatric labels and has direct implications for the design of mental health literacy and anti-stigma interventions. The research design and method may be of interest to those who wish to add to understanding of stigma through the use of experimental research, an option rarely used at present.

## Appendix

### Vignette scripts

Mark (vignette one- “well-adjusted”, does not meet DSM-IV criteria for Major Depressive Disorder)

“Hi I’m Mark and I’m around the same age as you. The past few months have been pretty good for me, on the whole. I’ve enjoyed hanging around with my friends after school and on weekends. I used to be pretty athletic but I gave up sports last year. I’m going to get back into it though. It’s nice not having as much academic work this year and I’m trying out different things. There’s a school show coming up and I’m going to audition for it. I was texting a girl for the last few weeks and we met up a couple of times but then she called it off. I was a bit put out by it and in a bad mood for a couple of days but I’m feeling alright about it now. My friends were pretty good about it. Things are good at home too, I didn’t have to do as much around the house during exams last year but it’s back to normal now! Most evenings I watch TV downstairs or see if anyone is around to hang out.”

Paul (vignette two- “the blues”, does not meet DSM-IV criteria for Major Depressive Disorder)

“Hey, I’m Paul and I’m about the same age as you. The last few months have been grand on the whole. I enjoy not having as much academic work this year. However, I feel a bit less motivated than I expected to try out new things. I was going to take up a new sport or volunteer more but I keep putting it off, but I’m going to get onto it soon! I feel being inactive makes it a bit harder to pay attention in class but I can concentrate well if I try and I’m still doing fine in school this year. There have been one or two days in the last month where I’ve felt a bit low and pessimistic, which is unlike me, but I’ve slept on it and it seemed better the next morning. I wasn’t able to put my finger on what exactly was getting me down and didn’t feel like being around people those days. But everything is fine with my friends and at home. Maybe it’s just part of being a teenager. Anyway, overall I’m ok at the moment.”

Killian (vignette 3- moderately impaired, meets DSM-IV criteria for Major Depressive Disorder)

“I’m Killian and I’m about your age. Last year I was getting on fine but recently I have found myself losing interest in a lot of things. For example I used to love watching films but I rarely find them enjoyable anymore. I also find I can’t get to sleep most nights and I then feel less energetic in school. It’s harder to stay focused on what the teacher is talking about than it used to be but if I really try I can keep up. Once or twice I have pretended to be sick and haven’t gone in. I think a few of my friends are picking up on it but so far I have denied it to them. I have skipped the odd training too but I don’t like letting the lads down so I still try to go, but I don’t care if we win our matches. I think my Mum and Dad are worried that I spend more time in my room in the evenings, sometimes thinking about whether life is worth living. Some nights I still meet up with friends but last week they brought this up and I found myself just making little of what’s going on and telling them not to worry.”

Simon (vignette 4- severely impaired, meets DSM-IV criteria for Major Depressive Disorder)

“My name is Simon, I’m around the same age as you. In the last couple of months I’ve been feeling sad and empty every day. I find it really difficult to feel interested in things I know I used to like. I’ve completely stopped going to training and ignore texts from my coach. I don’t see the point in going and don’t think I’d enjoy it anymore. I have trouble falling asleep at night and when I finally get to sleep, I find it very difficult to get up the following morning. I don’t enjoy food as much as I used to and when I’m hungry I often eat junk food. I have missed a lot of school recently and when I am in class, it’s difficult to concentrate, or to care about school work anymore. I don’t think I’m good at anything anymore. My friends in school are worried about me but I don’t want to talk about this. I feel an overwhelming loss of energy and regularly feel restless. I know my parents are also worried about me, they are being really nice, but I don’t feel like talking to them either and at home, I spend most of my time in my room, often on my laptop. I constantly feel guilty, but almost every day, I think that life is not worth living anymore.”
